# Identification and characterization of Dlc1 isoforms in the mouse and study of the biological function of a single gene trapped isoform

**DOI:** 10.1186/1741-7007-8-17

**Published:** 2010-03-03

**Authors:** Mohammad G Sabbir, Nichola Wigle, Shauna Loewen, Yuan Gu, Cordula Buse, Geoffrey G Hicks, Michael RA Mowat

**Affiliations:** 1 Manitoba Institute of Cell Biology, CancerCare Manitoba, 675 McDermot Avenue, Winnipeg, MB R3E 0V9, Canada; 2 Department of Biochemistry & Medical Genetics, University of Manitoba, Winnipeg, Canada

## Abstract

**Background:**

The Dlc1 (deleted in liver cancer 1) tumour suppressor gene codes for a RhoGTPase activating protein that is found inactivated in many tumour types. Several transcriptional isoforms have been described but the functional significance and tissue distribution of each form is presently poorly understood. Also, differences in the number of isoforms and splice variants reported still exist between different mammalian species. In order to better understand the number and function of the different variants of the Dlc1 gene in the mouse, we have carried out a detailed analysis. Extensive 3' RACE experiments were carried out in order to identify all possible Dlc1 isoforms and splice variants in the mouse. In addition, we have generated a gene trapped mouse that targets one of these isoforms in order to study its biological function. The effect of this gene trap insertion on the splicing of other isoforms has also been studied.

**Results:**

In addition to the known 6.1 and 6.2 Kb transcripts of Dlc1, our study revealed the existence of a novel 7.6 Kb transcriptional isoform in the mouse, which corresponds to the human 7.4 Kb (KIAA1723) cDNA transcript. A gene trapped embryonic cell line, with an insertion between Exon 1 and 2 of the 6.1 Kb transcriptional isoform, was used to generate a transgenic mouse. This line showed a significant reduction in the expression of the trapped isoform. However, reduced expression of the other isoforms was not seen. Mice heterozygous for the gene trapped allele were phenotypically normal, but homozygous mutant embryos did not survive beyond 10.5 days post coitum. Dlc1^gt/gt ^embryos showed defects in the brain, heart, and placental blood vessels. Cultured serum-free mouse embryo cells from Dlc1 deficient embryos had elevated RhoA activity and displayed alterations in the organization of actin filaments and focal adhesions. The Dlc1 deficient cells also exhibited increased wound closure in an *in vitro *scratch assay.

**Conclusions:**

The mouse has three major transcriptional isoforms of the Dlc1 gene that are differentially expressed in various tissues. A mouse with exon 1 of the 6.1 Kb transcript gt resulted in hypomorphic expression of Dlc1 protein and an embryonic lethal phenotype in the homozygous condition, which indicates that this isoform plays a major role in mouse development. The Dlc1 deficient cells showed altered cytoskeleton structure, increased RhoA activity and cellular migration.

## Background

The Rho family of GTPases control several important cellular processes, such as cell shape, motility, division and survival through a series of biochemical networks [1,2]. RhoGTPases have long been implicated in tumourigenesis, as Rho activity is found to be increased in transformed cells and is necessary for the transformation by the Ras oncogene [3,4]. The Rho proteins act as molecular switches, cycling between an active GTP bound state and an inactive GDP-bound state [5]. The RhoGTPase activating proteins (RhoGAPs) down regulate Rho by stimulating its intrinsic GTPase activity [6]. A significant number of RhoGAPs have been shown to have altered levels in a variety of human tumours and cell lines [7].

The deleted in liver cancer 1 (Dlc1) gene encodes a RhoGTPase activating protein (RhoGAP) that has been found to be inactivated by deletion or promoter methylation in many tumours, resulting in alterations in cellular proliferation, cytoskeleton reorganization and gene expression in tumour cells [8-15]. A recent study using representational oligonucleotide based microarray analysis showed that heterozygous deletion of Dlc1 occurred in ~50% of liver, breast and lung tumours and over 70% of colon cancers [10,16].

The Dlc1 RhoGAP is a multi-domain protein that contains a sterile alpha motif 2 (SAM2) interaction domain and a StAR-related lipid-transfer (START) domain [2,8,17-20]. The Dlc1 gene shows multiple transcription start sites and alternative splicing. In humans, three major transcriptional isoforms of the Dlc1 gene has been reported (for a review see [20]). The predominant 6.3 Kb transcript of Dlc1 encodes a protein of 123 KDa [11]. The existence of proteins for the human mRNA isoforms of 3.7 Kb (AK025544) and 7.4 Kb (KIAA1723) have recently been verified by Ko *et al*. [21]. In mouse tissues, two principal transcripts of 6.5 and 5.5 Kb and a minor transcript of 7.6 Kb have been reported by Northern blotting [22], but the complete characterization and tissue specific expression of all the isoforms and splice variants of Dlc1 gene in the mouse has not yet been carried out.

In order to better understand the biological function of various isoforms of Dlc1, we have generated a mouse with one of its isoform variants targeted. We have also characterized the different transcriptional isoforms and their tissue specific expression in the mouse. The effect of this gene trap insertion on the other variants has also been studied.

## Results

### Identification, characterization and tissue specific expression of mouse Dlc1 mRNAs and protein

**Figure 1:**
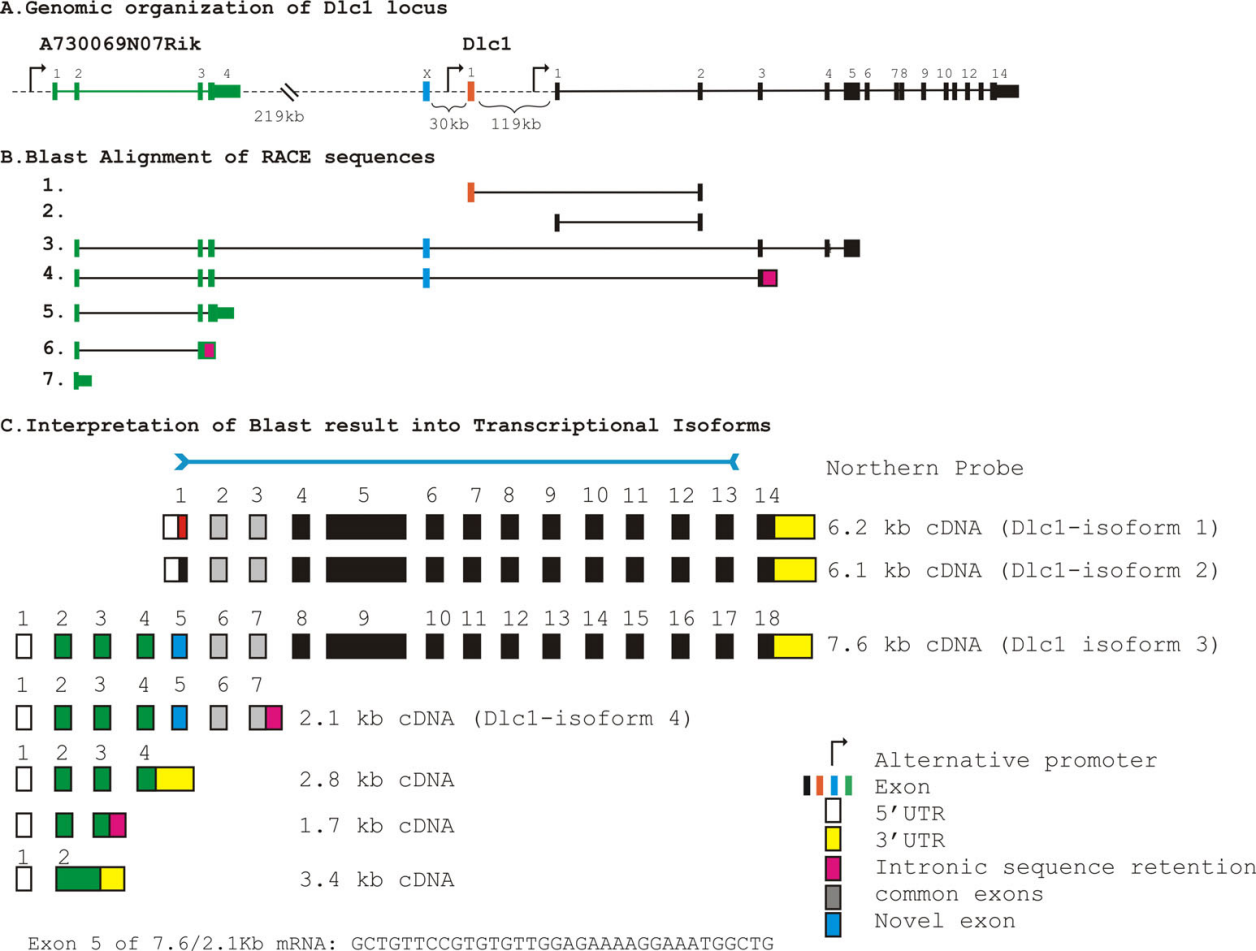
**Splicing pattern of the transcriptional isoforms of Dlc1 gene**. (A) Genomic organization of Dlc1 gene. Solid black rectangles represent exons of the Dlc1 gene; green rectangles represent exons of the unknown gene (A730069N07Rik). (B) Blast alignment of the 3'RACE sequences indicating the presence of 7.6 Kb and 2.1 Kb transcriptional isoforms in the UCSC genome database. (C) Predicted isoforms and splice variants of the Dlc1 gene based on the 3'RACE sequences and cDNAs in the databases. The exonic units are represented by solid rectangles separated by blank space which indicates an intron.

Extensive 3' RACE experiments were carried out in order to determine all possible isoforms and splice variants of the Dlc1 gene in the mouse (Figure 1). Transcripts 1 and 2 correspond to the two previously described transcripts of 6.2 and 6.1 Kb, which agrees with the current major translated transcripts (isoform-1 and 2, respectively) found in the Vega database (Version 3, 02/06/2008; Figure 1B). These two transcripts are controlled by two alternative promoters (Figure 1A). A previously described 2.8 Kb upstream transcript (**AK053142**) had not been linked to Dlc1 in the mouse; however, it shows partial homology to the human Dlc1 7.4 Kb transcript. We therefore included a primer for this transcript in our 3' RACE experiments. The regions of homology of transcripts 3 and 4 to the Dlc1 locus are shown in Figure 1B. These results show a linkage of this upstream 2.8 Kb transcript with exon 2 of Dlc1, including a novel exon not previously reported. Based on these complimentary DNAs (cDNAs) and others, found in the databases, we predict that there is a 7.6 Kb transcript, which arises from a promoter greater than 220 Kb upstream from exon 1 of the 6.2 Kb Dlc1 transcript (Figure 1C). This 7.6 Kb transcript (GenBank accession No. GU1280500, isoform-3) contains sequences in common with the previously reported 2.835 Kb cDNA (AK048960). A second 2.1 Kb transcript (GenBank accession No. GU1280389, transcript-4) starting from the same promoter retains 160 bp of intronic sequence downstream of exon 3 of the 6.1 Kb transcript (Figure 1B and 1C). In addition, transcripts of 3.4, 2.8 and 1.7 Kb (Nos. 5, 6 and 7) from this same upstream promoter, that do not link to the Dlc1 gene, were also found (Figure 1B and 1C, GenBank accession No. GU1280505).

**Figure 2:**
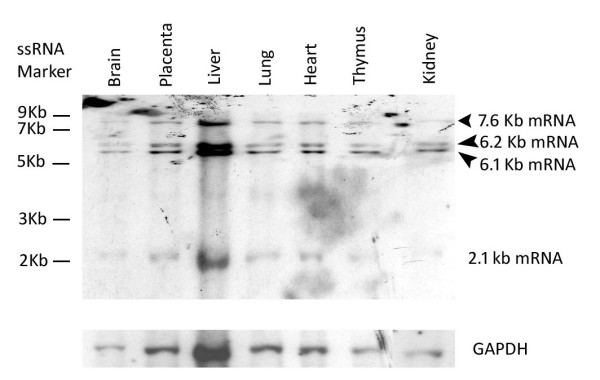
**Northern blot of ribonucleic acid (RNA) from different mouse tissues**. Poly(A)^+ ^RNA from different mouse tissues were transferred to a nylon membrane and hybridized sequentially with a 3.3 Kb deleted in liver cancer 1 RNA probe and a mouse GAPDH probe.

To confirm these transcripts, northern blotting was carried using a 3.3 Kb ribonucleic acid (RNA) probe that contained exon 1 to 13 of the 6.1 Kb Dlc1 transcript (Figure 2). This probe revealed the existence of transcripts (7.6, 6.1, 6.2 and 2.1 Kb) in all the tissues examined. However, the 7.6 Kb splice variant was most prominent in the liver and placenta and appeared to be a minor transcript in other tissues.

**Figure 3:**
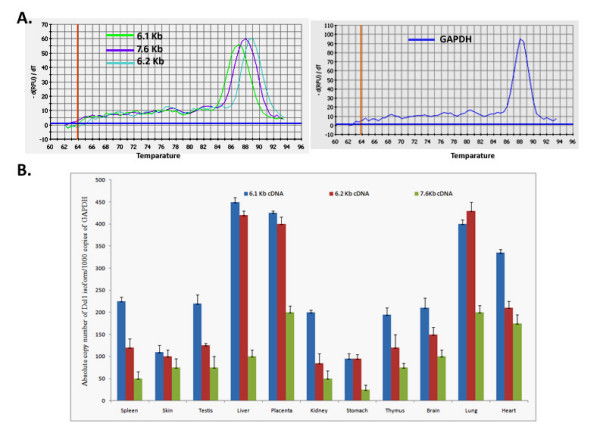
**Absolute quantification of Dlc1 transcriptional isoforms in mouse tissues by real time reverse transcription polymerase chain reaction (PCR)**. (A) Representative melt curve analysis plot of the PCR amplified product specific to Dlc1 isoforms and GAPDH. (B) Copies of Dlc1 6.1, 6.2, and 7.6 Kb transcriptional isoforms per 1000 copies of GAPDH (absolute copy number of cDNA). The mean and standard deviation was determined from triplicate experiments.

Real time reverse transcription polymerase chain reaction (RT-PCR) was carried out in order to quantify the relative expression of the transcripts in various tissues (Figure 3). These results reveal that the Dlc1 isoforms are expressed abundantly in all the mouse tissues that we examined, which agrees with the data observed by northern blot analysis (Figure 2). However, using real time RT-PCR analysis, the 6.1 Kb transcript seems to be expressed highest in the liver, followed by the placenta, lung, heart, spleen, testis, brain, kidney, thymus and the skin. The 6.2 and 7.6 splice variants were expressed at lower levels in the other organs compared with the 6.1 Kb isoform. Liver and placenta appears to predominantly express the 6.1 and 6.2 Kb transcripts whereas the 7.6 Kb transcript was predominantly expressed in lung, heart and placenta based on real-time RT-PCR, in agreement with the northern blot.

**Figure 4:**
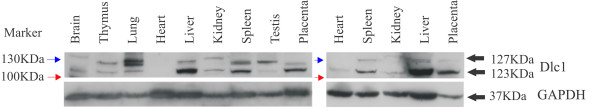
**Dlc1 protein expression in different mouse tissues**. Western blots of total cellular protein extracted from different mouse tissues using Dlc1 polyclonal antibody. The upper blot shows expression of Dlc1 related proteins and the lower blot shows the GAPDH loading control.

In order to determine the amount of the Dlc1 protein in different tissues, we carried out western blotting using a polyclonal anti-Dlc1 antibody that was raised against a peptide containing amino acids residues 111-370 from exon 5 of the 6.1/6.2 Kb transcripts (isoforms 1 and 2, Figure 4). This antibody should pick up proteins translated from the 6.1, 6.2 and 7.6 Kb transcripts as Exon 5 is common to all three. The Dlc1 antibody detected a 123 KDa protein presumably derived from the 6.1 Kb transcript. The Western blot revealed a very high expression of 123 KDa Dlc1 protein in the liver, placenta and spleen. The molecular weight of the Dlc1 protein derived from the 6.2 Kb transcript is predicted to be 127 KDa. In the same western blot test we found a band of approximately 127 KDa, which showed a high expression in the thymus, lung, testis, spleen and liver (Figure 4). Sometimes a doublet of the 127 KDa band was seen, which suggests a possible post-translational modification. Further experiments will be needed in order to confirm the identity of the 127 KDa band and the other cross reacting proteins. With this antibody, we were not able to detect any proteins in the region of 169.8 KDa - the predicted size of the protein from the 7.6 Kb transcript (data not shown). The expression profile of Dlc1 isoforms among different tissues were correlated, to some extent, with the quantitative PCR and the northern blot.

### Methylation status of the alternative promoters of Dlc1 6.1 and 6.2 Kb transcripts

**Figure 5:**
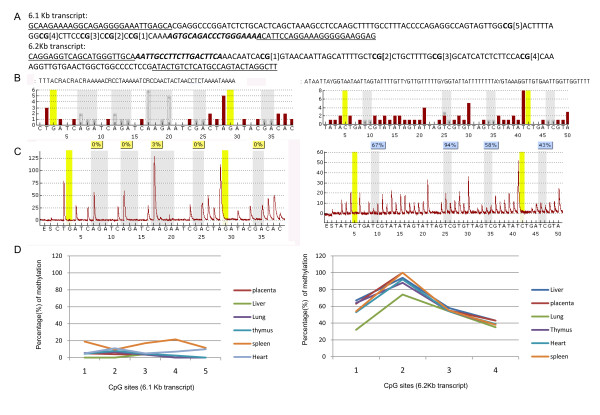
**Methylation status of the Dlc1 alternative isoform promoters in different mouse tissues**. (A) The Dlc1 6.1 and 6.2 isoform promoter sequences showing the location of the polymerase chain reaction primers (underlined), sequencing primer (bold and italics) and the CpG islands (numbered) that were analysed. (B) The reference peak heights for the 6.1 (left) and 6.2 Kb (right) isoform promoter sequences. (C) Pyrograms for liver DNA, showing the 6.1 Kb isoform promoter (left) and the 6.2 Kb isoform promoter (right). (D) The mean percentage of methylation for the different CpG islands in the mouse tissues examined, 6.1 Kb promoter (left), 6.2 Kb promoter (right).

Since the 6.1 and 6.2 Kb transcripts showed differential tissue expression, we wanted to determine if this was reflected by differences in promoter methylation. We used bisulphite treatment of DNA followed by PCR and pyrosequencing to study the promoter methylation status of the 6.1 and 6.2 Kb transcripts in various tissues. For our studies, we targeted five CpG islands located 502 bp upstream of the start codon for the 6.1 Kb transcript and four CpG islands located 851 bp upstream of the start codon of 6.2 Kb transcript (Figure 5A). The pyrosequencing revealed much lower methylation of the 6.1 Kb promoter region (1-19% mean methylation) in the 5 CpG islands; the 6.2 Kb promoter region showed a higher percentage of mean methylation of 60-65% in all the tissues examined (Figure 5D).

### Characterization of the mice heterozygous for the gene trap allele

**Figure 6:**
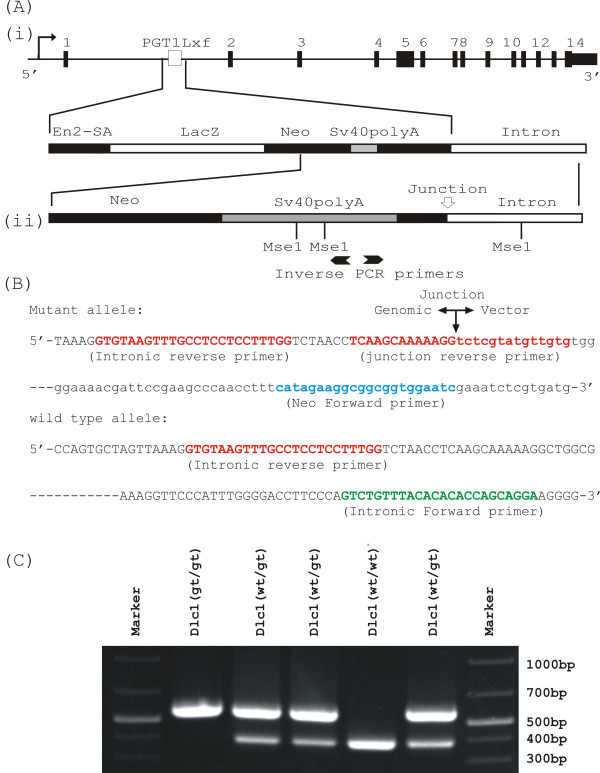
**Genotyping of the Dlc1 gene trapped mouse**. (A)-(i) Diagrammatic representation of the Dlc1 locus showing the insertion site of the gene trap vector pGT1Lxf; (ii) the gene map of the integrated vector. (B) Sequence of the gene trap vector insertion site with the intronic junction fragment. The polymerase chain reaction (PCR) primers sequences used to genotype the gene trap mutant mouse are highlighted. (C) Multiplex PCR showing homozygous, heterozygous and wild type bands in 10.5 dpc Dlc1 embryos.

Since there were several embryonic stem cell lines available with exon 1 of the 6.1 Kb transcript gene trapped (gt), we made a mouse using an embryonic cell line, named XE082, to assist the functional characterization of this isoform. We determined the integration site of the promoter trap plasmid in this line by inverse PCR and used this information for genotyping the transgenic mice made with this line (Figure 6). Adult mice heterozygous for the Dlc1 gene trap did not show any gross physical, histological or behavioural abnormalities. As well, we have not seen any spontaneous tumours in heterozygous mice living to at least 2 years of age.

**Figure 7:**
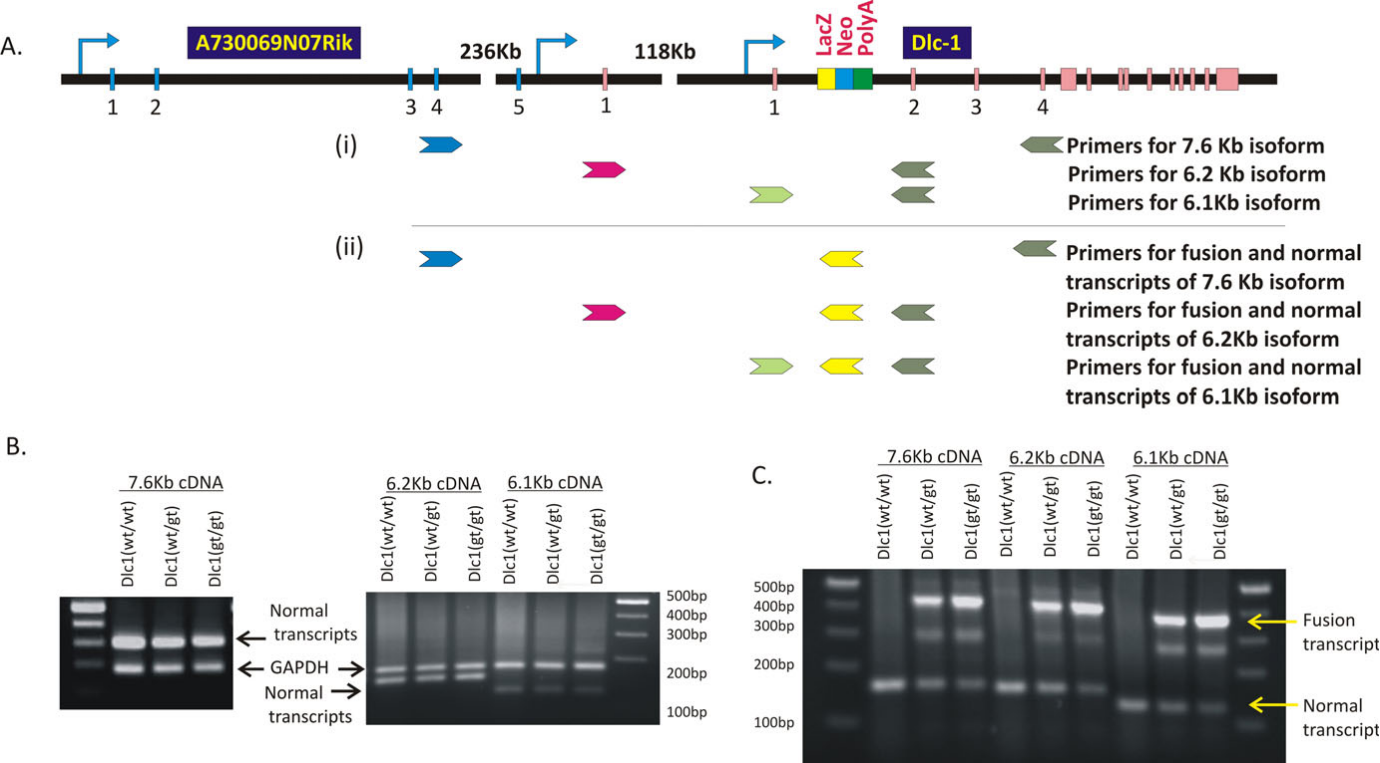
**Multiplex reverse transcriptase polymerase chain reaction (RT-PCR) showing the effect of gene trap insertion on the various Dlc1 isoforms**. (A) Diagrammatic representation of the Dlc1 gene showing the position of different PCR primers sets used; (i) to show relative expression of GAPDH mRNA and Dlc1 isoforms and (ii) to show the relative expression of gene trap fusion transcripts and Dlc1 normal transcripts. (B) Multiplex RT-PCR showing the expression of 7.6 Kb, 6.2 Kb and 6.1 Kb Dlc1 isoforms and alternative splice forms relative to GAPDH expression (C) Multiplex RT-PCR showing the expression of 7.6 Kb, 6.2 Kb and 6.1 Kb Dlc1 isoform normal transcripts relative to their fusion splice products with βgeo of the gene trap vector.

**Figure 8:**
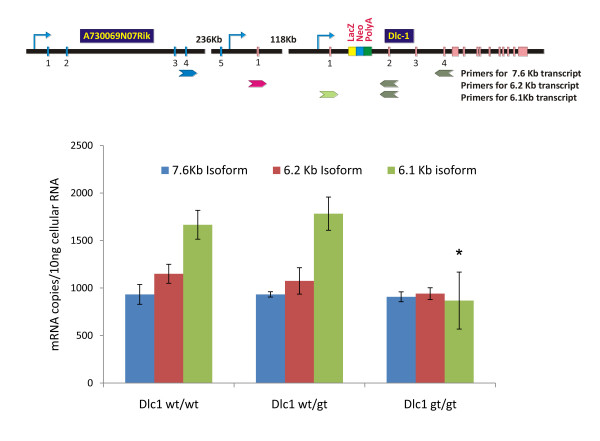
**Quantitative real-time reverse transcriptase polymerase chain reaction (RT-PCR) analysis of the absolute copy number of Dlc1 isoforms in Dlc1 deficient serum-free mouse embryo cell lines**. The mean and standard deviation was determined from triplicate experiments. The upper panel of the diagram shows the position of the RT-PCR primers used for amplification of the specific isoforms. *P < 0.001, by one way ANOVA test.

Multiplex RT-PCR (Figure 7B) and real time RT-PCR analysis (Figure 8) showed a reduction of the 6.1 Kb transcript in the homozygous gt serum free mouse embryonic cell lines (SFME) compared with wild type and heterozygous lines. The 6.2 and 7.6/2.1 transcripts showed only a slight reduction, despite the fact that all three transcripts can be spliced into the βgeo reporter cassette (Figure 7C). These results indicate that the gene trap's main effect resulted in hypomorphic expression the 6.1 Kb isoform.

### Abnormalities in the Dlc1^gt/gt ^embryos

**Figure 9:**
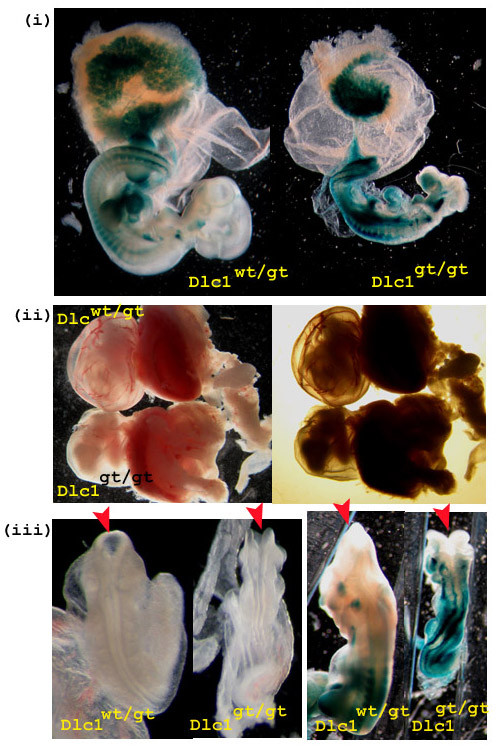
**Dlc1^gt/gt ^embryos**. (i) X-gal stained Dlc1^wt/gt ^and Dlc1^gt/gt ^embryos and placenta showing expression of Dlc1 exon1-βgeo fusion protein. (ii) Impairment of yolk sac blood vessel formation in Dlc1^gt/gt ^mouse embryos. The yolk sacs of 10.5 dpc Dlc1^wt/gt ^and Dlc1^gt/gt ^mouse embryos with the later, showing no detectable blood vessels. (iii) The Dlc1^gt/gt ^embryos showing failure to close the neural tube at the anterior part of the body (left: unstained embryos and right: X-gal stained embryos).

**Figure 10:**
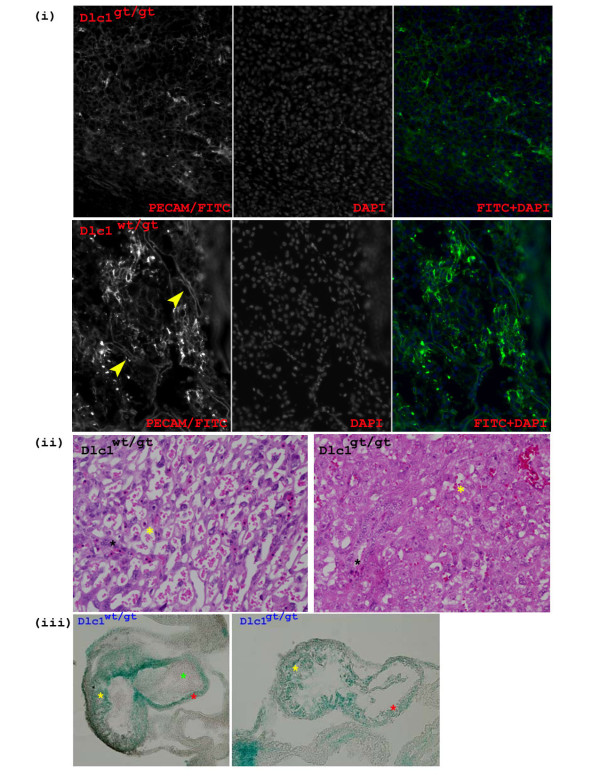
**Placental and heart abnormalities in Dlc1^gt/gt ^embryos**. (i) Immunofluorescent staining of the placenta of 10.5 days post coitum (dpc) Dlc1^wt/wt ^and Dlc1^gt/gt ^embryos with PECAM (CD31) antibody. Twelve micrometer thick frozen paraformaldehyde fixed placental sections were incubated with anti-PECAM-1 antibody and then with FITC conjugated secondary antibody. (ii) The yellow arrow indicates a continuous network of blood vessel in the placental labyrinth haematoxylin and eosin stained sections of placental labyrinth of 10.5 dpc Dlc1^wt/gt ^and Dlc1^gt/gt ^embryos. Black asterisks indicate fetal erythrocytes and the yellow asterisk indicates maternal red blood cells. (iii) Transverse section of the X-gal stained heart of 10.5 dpc Dlc1^wt/gt ^and Dlc^gt/gt ^embryos. The yellow asterisk indicates normal thick trabeculated ventricular wall; the red asterisk thin smooth atria wall; and the green asterisk with foetal erythrocytes.

No mice homozygous for the gt Dlc1 allele were observed at birth from heterozygous intercross matings. Histological analysis of 10.5-11.5 days post coitum (dpc) embryos from heterozygous matings showed that Dlc1^gt/gt ^embryos were necrotic and displayed growth retardation that varied in severity (Figure 9i). Most of the embryos had a retarded brain structure and an abnormally enlarged heart (Figure 9i). The presence of the βgeo cassette in the gene trap vector allowed for expression analysis of the Dlc1 gene after X-gal staining. The anterior neural tube was open and contorted in the homozygous gene trapped embryos (Figure 9iii). X-gal stained hearts showed thin and underdeveloped atrial and ventricular walls with an apparent lack of blood cells inside the heart (Figure 10iii). The homozygous gt mice also showed an absence of blood vasculature in the yolk sac (Figure 9ii) and in the placental labyrinth (Figure 10i &10ii). The haematoxylin and eosin (H&E) stained sections of Dlc1^wt/gt ^placental labyrinths at 10.5 dpc showed sinusoids with maternal enucleated red blood cells (RBC) and embryonic blood vessels with nucleated fetal erythrocytes in close proximity with each other (Figure 10ii). In contrast, the embryonic and placental vasculature was severely reduced in the Dlc1^gt/gt ^placenta, as revealed by immunofluorescent staining with the endothelial and haematopoietic cell marker CD31 (Figure 10i). H&E staining of the Dlc1^gt/gt ^placenta also showed aggregates of erythrocytes in collapsed blood vessels (Figure 10ii). X-gal staining of the 10.5 dpc homozygous gt embryos revealed a strong expression of the beta-galactosidase fusion protein in the anterior part of the notochord, somites, liver, heart, lung, forebrain, placenta and tail bud (Figure 9i &9iii). The defects in placenta and the vitelline blood vessels are probably the cause for the embryonic lethal phenotype of the homozygous gene trapped embryos.

### Reduced level of Dlc1protein in a serum free Dlc1^gt/gt ^mouse embryos cells

**Figure 11:**
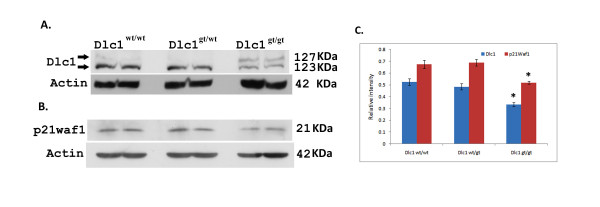
**Dlc1 and p21^WAF1 ^protein expression in Dlc1^wt/wt^, Dlc1^wt/gt^, and Dlc1^gt/gt ^SFME cell lines**. Western blots of total cellular protein extracted from different serum-free mouse embryo (SFME) cell lines. (A) Upper blot shows the expression of Dlc1 protein and lower blot shows actin expression. (B) Upper blot shows the expression of p21^WAF1 ^protein and the lower blot shows actin expression. (C) Plot showing the relative intensity of Dlc1 123 KDa protein and p21^WAF1 ^protein in SEME cell lines. Means and standard error of mean determined from at least six independent experiments using cell lines derived from different mouse embryos are given. * *P *< 0.001, by one way ANOVA test.

Dlc1 protein expression was determined in serum free mouse embryo cell lines (SFME) derived from 9.5 dpc embryos. The homozygous Dlc1^gt/gt ^SFME cells showed significantly reduced Dlc1 protein levels (*P *< 0.001) in western blots compared with the heterozygous or wild type cells (Figure 11). We found specific reduction of the 123 KDa band corresponding to the predicted size of the 6.1 Kb transcript of Dlc1. There was also an increase of a cross reacting 127 KDa band in the homozygous gt cells when compared to the wild type and heterozygous lines. This protein size corresponds to the predicted size for the 6.2 Kb transcript.

### Dlc1^gt/gt ^SFME cells showed increased RhoA activity and an altered cytoskeleton along with increased cell motility

**Figure 12:**
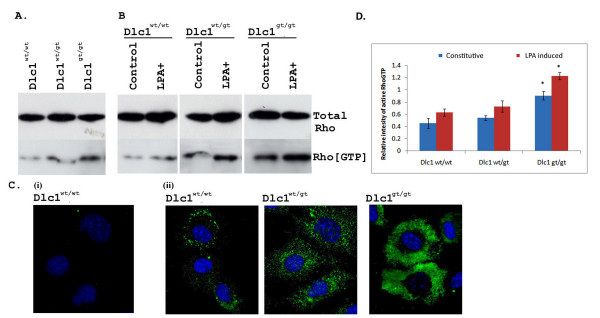
**RhoGTP pull down assay and *in situ *RhoGTP affinity assay**. RhoGTP pull down assay showing (A) constitutive and (B) LPA induced and control levels of active RhoGTP in Dlc1^wt/wt^, Dlc1^wt/gt ^and Dlc1^gt/gt ^SFME cell lines. (C) Dlc1 SFME cells exponentially growing on glass slides were fixed and cells were incubated with 50 μg/mL of soluble GST (i) or GST-RBD (ii). RhoGTP-bound RBD was detected by GST immunostaining. (D) Plot showing the RhoGTP to total Rho protein by scanning the relative intensity of the protein bands in non-treated and LPA induced in SEME cell lines. Means and standard error of mean determined from at least six independent experiments using cell lines derived from different mouse embryos are given. *P < 0.001, by two way ANOVA test.

If Dlc1 activity is reduced in the gene trapped cells, one would expect to see an increase in active Rho activity. The active Rho levels in the Dlc1 deficient SFME cell lines were measured by a Rho-GTP pull-down assay. The Dlc1^gt/gt ^cells showed a significantly higher (*P *< 0.001) constitutive level of active GTP bound RhoA (Figure 12A, B and 12D). The induction of active Rho following LPA treatment was also significantly higher (*P *< 0.001) in the Dlc1^gt/gt ^cell line compared with the Dlc1^wt/wt ^or Dlc1^wt/gt ^lines. Since RhoA has been implicated in podosome formation in osteoclasts [23] and dendritic cells [24], we wanted to see if the subcellular localization of endogenous active RhoA was altered using an *in situ *Rho-GTP affinity assay. As in the active pull down assays, increased levels of active RhoA was detected by GST-Rho Binding Domain (RBD) protein staining in the Dlc1^gt/gt ^cells when compared with the wild type and heterozygous cells (Figure 12C).

**Figure 13:**
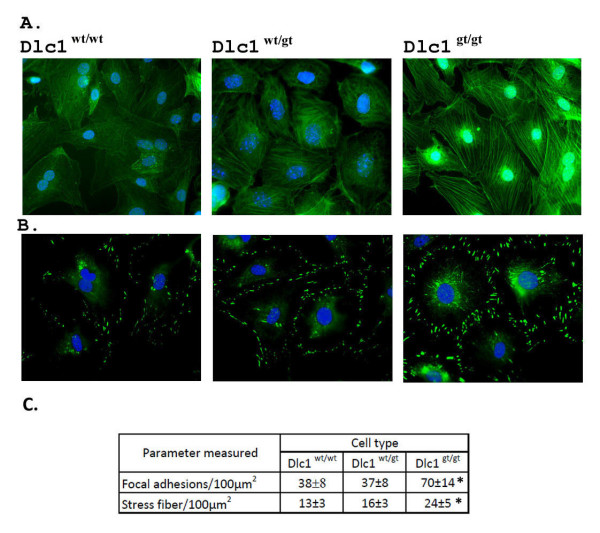
**Altered cytoskeletal organization in Dlc1 gene trapped and normal serum-free mouse embryo (SFME) cell lines**. (A) SFME cell lines were stained with phalloidin-TRITC and DAPI. (B) Localization of vinculin in SFME cell lines by immunofluoresence. Cells were also stained with DAPI. (C) Cells were analysed to determine total cell area and the numbers of focal adhesions and stress fibres present as outlined in materials and methods. Means and standard error of mean determined from at least six independent experiments are given. *P < 0.001, by one way ANOVA test.

The formation of actin stress fibres and focal adhesions were compared among the various SFME lines detected by fluorescent conjugated phalloidin and anti-vinculin antibody staining (Figure 13A, B and 13C). The Dlc1 gt cells showed a significant increase (*P *< 0.001) in actin stress fibre formation and increased vinculin associated focal adhesions, when compared with the wild type and heterozygous cell lines.

It has been previously reported that active RhoA activity can down regulate p21^WAF1 ^expression [25]. The Dlc1^gt/gt ^cells also showed significantly reduced (*P *< 0.001) constitutive levels of the p21^WAF1 ^protein in comparison to the cell lines derived from heterozygous and wild type embryos (Figure 11). We have also found a positive correlation between reduced expression of Dlc1 and p21^WAF1 ^in cell lines (*R *= 0.645; *P *= 0.004). The preceding results indicate that the Dlc1 gt cells showed increased RhoA activity.

**Figure 14:**
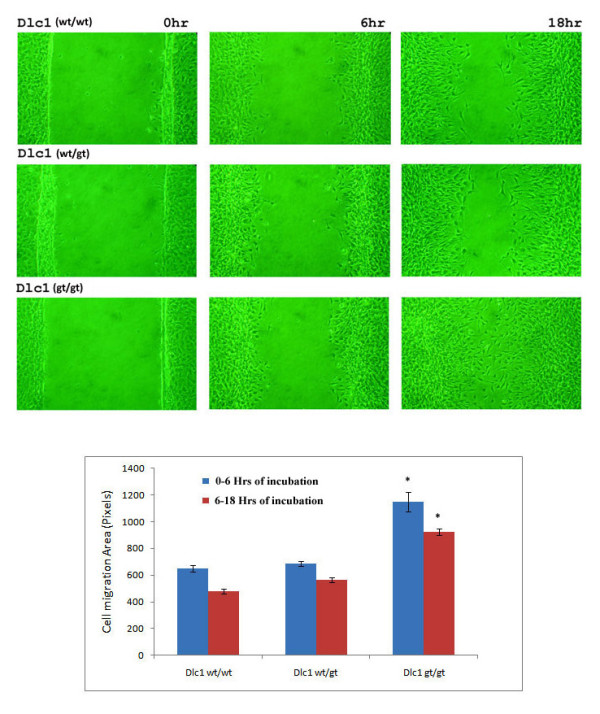
**Scratch assay of Dlc1 gene trapped and normal mouse embryonic fibroblast cell lines at various time points**. (A) The homozygous Dlc1^gt/gt ^cells show increased wound closure by 18 h compared with the Dlc1^wt/wt ^and Dlc1^wt/gt ^cell lines. (B) Scratch assays at different time points. The plots show the changes in wounded surface area after 6 h [Blue] and 18 h [Red] after the wound creation. Mean ± standard error of mean of three independent experiments for each cell type and eight areas imaged for each experiment, *P < 0.001, by one way ANOVA test.

Rho proteins are important players in the regulation of cell motility and reduced expression of Dlc1 enhances cell migration. In order to study the effect of hypomorphic expression of Dlc1 on directed cell migration, we performed scratch assays by wounding confluent monolayers of mouse embryonic fibroblast cell lines derived from wild type, heterozygous and homozygous embryos. The Dlc1^gt/gt ^cells showed more rapid wound closure that was significantly different compared to the rate of wound closure in wild type and heterozygous cells (*P *< 0.001; Figure 14), indicating increased cell motility. The amount of wound closure decreased slightly at longer times, possibly due to change in direction of cell movement.

## Discussion

Alternative splicing and promoter usage of genes allows for an expansion of protein diversity and control. Recent evidence suggests that 30%-50% of the human and mouse genes have multiple alternative promoters [26]. In humans, at least three transcriptional isoforms (6.3, 3.7 and 7.4 Kb length) of Dlc1 gene have been identified that are expressed under the influence of three alternative promoters spanning approximately 400 Kb. The mouse Dlc1 gene on chromosome 8B1 and the human gene on 8P22 have an almost identical exon/intron structure, except for the presence of an extra codon in exon 5 of the mouse [22,27]. In a previous study using 5' RACE and northern blotting analysis, Durkin *et al*. reported the presence of 3 Dlc1 transcripts in the mouse of 5.5, 6.5 and 7.5 Kb in length [22]. However, in our study using extensive 3' RACE and northern blot analysis, we have found four major transcriptional isoforms of Dlc1 (2.1, 6.1, 6.2 and 7.6 Kb). The complete characterization of the 7.6 Kb and 2.1 Kb transcripts lead to the identification of a novel exon of 34 bp in length, which connects the Dlc1 gene upstream of exon 2 to an additional 1.7 Kb of exon sequences, resulting in the 7.6 Kb fusion transcript. The upstream 1.7 Kb exon sequences were generated from a previously uncharacterized gene 5' of Dlc-1 (A730069N07Rik). It is interesting to note that comparison of the mouse 7.6 Kb mRNA with the human 7.5 Kb transcript revealed that the 5^th ^exon is exactly 34 bp in length and only differs in its relative position within the intronic sequences. The conserved nature of this novel exon sequence supports a functional significance for the 7.6 Kb mRNA. The human 7.4 Kb, KIA1723, cDNA has the potential to encode for a 1528 amino acid protein [28]. Recently Ko *et al*. have shown high expression of a greater than 150 KD protein in the human heart tissue using an antibody based on this cDNA sequence [21]. The putative promoter region of both the human and mouse Dlc1 7.6 Kb mRNA is approximately 400 Kb upstream of the exon 2 of the Dlc1 6.2 Kb transcript. Shorter transcripts originating from this promoter are also found, which do not encode Dlc1 RhoGAP related sequences. The existence of this larger Dlc1 polypeptide has not yet been verified in the mouse and we have not yet seen a protein of this size on our western blots. The mouse 2.1 Kb transcript retains only exon 2 and 3 of the regular Dlc1 transcripts and would not encode a RhoGAP related protein. Understanding the function of these shorter transcripts will have to await specific knockout mouse models. Analysis of the nucleotide sequence of the three isoforms reveals that these isoforms have a conserved domain structure and that they may be functionally redundant. However, these isoforms were not sufficient to rescue the embryonic lethal phenotype of our homozygous gt mice.

The gene fusions observed in the 7.6/2.1 Kb transcripts may be due to the bypassing of 3' end formation sites that has been observed for other genes in the Fantom database [29]. Exon 4 of the A730069N07Rik transcript contains two successive polyadenylation signals TGTTTT and AATAAA, which are considered weak in comparison to the presence of three successive polyadenylation signals (AATAAA, ATTAAA, and TGTTTT) [30] present in the 14th exon of regular Dlc1 transcripts. The weak polyadenylation signal may be responsible for bypassing the 3' end formation and lead to the formation of a fusion transcript between the genes. However, the conserved nature of the exon 5 of the 7.6 transcript contradicts this finding and further study is necessary in order to validate these observations.

The 6.1 Kb Dlc1 transcript is predicted to encode a 123 KDa protein, which is highly expressed in the forebrain, anterior tip of the notochord, somites, liver, heart and placenta, suggesting an important role for this Dlc1 isoform in the development of theses organs. The 6.2 Kb alternative transcript contains a novel first exon located 133 Kb upstream of exon 2 and potentially codes for a 127 KDa protein. The first exon of the 6.2 Kb transcript substitutes 47 aa of the first 13 aa of the 6.1 Kb Dlc1 transcript. The existence of the 127 KDa Dlc1 protein has not yet been fully validated. However, in our western blots, using an antibody raised against a peptide to exon 5, we identified a band equivalent to 127 KDa. The expression level of 127 KDa Dlc1 protein showed tissue specific differences from the 123 KDa protein. For example, testes showed expression of the 127 KDa band and reduced levels of the 123 KDa band (Figures 3 and 4). There is possibility that the 127 KDa protein is a cross reacting non-specific protein. However, we argue that the 127 KDa band is the protein product of the 6.2 Kb isoform because of its molecular weight, which almost exactly matches the predicted protein product of 6.2 Kb isoform. Another reason for such an argument is that the 127 KDa protein band has increased intensity in the Dlc1^gt/gt ^cell line as opposed to the wild type or heterozygous cell lines. This may be explained as a cellular response to complement the reduced level of 6.1 Kb transcript in the gt cells or an effect of the relative abundance of 6.2 kb isoform. The 6.1 and 6.2 Kb Dlc1 isoforms may be functionally redundant because of their structural resemblance and, thus, could complement each other. Recently, such functional redundancy has been shown by Ko *et al*. in a study using Myc tagged Dlc1 isoforms expressed in hepatoma cell lines [21]. They showed that human Dlc1 isoforms α and β (equivalent to mouse isoforms 1 and 3) could suppress stress fibre formation *in vitro*. However, further experimentation is necessary in order to test such a claim.

The N terminus of Dlc1 protein contains a sterile alpha motif (SAM)2 interaction domain, amino acids 13-64 in the 6.1 Kb and 47-98 in the 6.2 Kb transcripts. The SAM domain is a motif that occurs in many transcription factors and signalling proteins [19]. The 123 and 127 KDa Dlc1 proteins differ in exon 1, which is a short stretch of amino acids upstream of the SAM2 domain. Deletion of the SAM domain increases the velocity of cell migration but reduces directionality by acting as a negative regulator of Dlc1RhoGAP activity [31]. A truncated version of Dlc1 protein that contains the N terminal domain, but not the GAP domain, localizes to focal adhesions and can act as a dominant negative protein [32]. Recently, in hamster kidney cells, co-localization of Dlc1 protein with Caveolin-1 in focal adhesions was found to be independent of the SAM domain [33]. However, the Dlc1 SAM2 domain has been shown to interact with the elongation factor EF1A1, targeting it to the membrane periphery and membrane ruffles [34].

It is possible that exon 1 of the Dlc1 isoforms may interact with different proteins or allow different subcellular localization for the spatial regulation of cell movement. In that case, the 6.1 Kb and 6.2 Kb isoforms may not be able to rescue each other's RhoGAP activity due to different interacting partners or subcellular localization. Alternatively, the two isoforms may have different temporal or tissue specific expression during development, a theory which is supported by our results. Experiments that will test these possibilities are ongoing.

The mean frequency of the promoter methylation for the Dlc1 isoforms helps to explain the tissue expression pattern. The promoter of the 6.1 Kb transcript showed a significantly lower mean percentage of CpG methylation (1%-19%) compared with the promoter of 6.2 Kb transcript (62%-65%). Hypermethylation of the Dlc1 promoter is a common mechanism for transcriptional repression of the Dlc1 gene in several human malignancies and hypermethylation of the 6.1 Kb transcript promoter is usually found in human tumours [9,14].

The gene trap insertion occurred within intron 1 of the Dlc1 6.1 Kb transcript and this allowed us to examine the functional significance of the specific loss of this transcript. The gene trap vector inserted in the Dlc1 locus should delete the majority of the downstream coding sequences if obligatory cleavage and polyadenylation at the SV40 polyadenylation signal of the gene trap construct occurs. By multiplex PCR, we showed the existence of fusion transcripts for all three of the Dlc isoforms spliced to the βgeo coding region. However, only the 6.1 Kb transcript was significantly reduced, resulting in the hypomorphic expression of the 123 KDa protein and subsequent phenotypic abnormalities in the mouse. The hypomorphic expression was presumably due to low level splicing around the gene trap plasmid insert. There is a possibility that the phenotypic abnormalities in our study could be due to the disruption or deletion of a microRNA expressed from this intron [35]. However, we have not found any evidence of any known microRNAs anywhere near or within this intron of Dlc1 in the miRBASE database, as well as any computationally identified candidate microRNA genes[36,37]. Also, our gene trapped mouse phenocopies the Dlc1 exon 5 knockout mouse of Durkin *et al*. [38]. These results suggest that the gt ES cell libraries maybe a useful resource for studying specific knockdown of transcriptional isoforms and splice variants of genes.

The Dlc1 gene plays a critical role in regulating cellular functions during early murine development. The abnormalities found in the Dlc1 gt embryos are a common occurrence in mouse gene knockouts that result in death at the early stages of embryogenesis, due to organ systems failure [38,39] and are often associated with cardiac or placental defects [38]. The abnormally enlarged heart, lack of yolk sac blood vessels and underdeveloped blood capillaries in the placental labyrinth of the homozygous Dlc1 gt embryos appeared at a developmental stage when the Dlc1 transcripts are normally expressed and, most probably, caused the embryonic lethality. Whether the heart and brain defects are a direct result of Dlc1 loss or a consequence of the lack of placental blood vessel formation will have to await the isolation of conditional Dlc1 knockout mice.

Previously, it has been shown that targeted disruption of mouse Rho associated coiled coil kinase-2 (ROCK-II) gene resulted in intrauterine growth retardation and defective branching morphogenesis of the placental labyrinth [40]. The ROCK-II mRNA has been found to be expressed abundantly in placenta [41]. The active form of RhoA binds to ROCK kinase, which phosphorylates myosin phosphatase, resulting in increased myosin light chain (MLC) phosphorylation allowing contraction [41]. The Rho/ROCK signalling pathway also contributes to the Ca^2+ ^sensitization of smooth muscle contraction [42]. RhoA is abundantly expressed in vascular smooth muscle and its activation increases vascular tone, resulting in increased permeability [43]. Rho GTPases has long been implicated in the regulation of endothelial permeability [44]. Recently, it has been shown that increased expression of constitutively active RhoA induced cell rounding and apoptosis of endothelial cells [45]. Therefore, it is possible that Dlc1 protein plays a significant role in placental and heart development due to its regulatory effect on Rho/ROCK signalling pathway.

The Dlc1 protein has been reported to be localized to the focal adhesions as shown by colocalization with the focal adhesion protein vinculin [46]. Focal adhesions are a structural link between the actin cytoskeleton and the extracellular matrix and are regions of signal transduction that can regulate growth control [47-49]. We have shown that the formation of actin stress fibres and vinculin rich focal adhesions were significantly increased in the hypomorphic Dlc1^gt/gt ^SFME and fibroblast cell lines. Dlc1 has been implicated in the phosphoinositide and RhoGTPase signalling pathways, both of which can regulate stress fibre formation and focal adhesions assembly [50-52]. Similar findings have been reported by Holeiter *et al*. in breast cancer cells using siRNA mediated knockdown of Dlc1 transcripts [53] and Xue *et al*. in embryonic liver progenitor cells using shRNA [10]. In contrast to our results, Durkin *et al*. reported that their knockout mice showed reduced actin stress fibre and vinculin rich focal adhesions in their Dlc1^-/- ^mouse embryonic fibroblast (MEF) cells [38]. A possible reason for this may be that the culturing of our SFME cells on fibronectin coated plates, may stimulate the Rho signalling allowing the formation of stress fibres. Using soluble GST-RBD in order to detect endogenous RhoGTP, we have been able to show that the Dlc1^gt/gt ^cells exhibit increased RhoA activity and subcellular localization of active RhoA at the focal adhesions. Similar findings have been previously reported by Berdeaux *et al*. in nontransformed fibroblasts [54]. Rho proteins are important regulators of cellular migration [55]. In addition to the cytoskeletal changes, the Dlc1^gt/gt ^MEF cells showed significantly increased cellular migration in wound closure assays compared with the heterozygous or wild type MEFs. Similarly, Holeiter *et al*. found rapid wound closure in scratch assays using RNAi to knockdown Dlc1 in MCF7 breast cancer cells [53]. The cellular migration rate decreased as the cells approached to the midline of the wound from both sides, which might be because of changes in the direction of migration due to contact or the depletion of nutrients in the media. All of this evidence is consistent with the suggestion that Dlc1 deficiency may be a driving force for cellular migration by remodelling the actin cytoskeleton and focal adhesions.

Recently, it has been shown that in H-Ras oncogene transformed fibroblasts, RhoA may cooperate in transformation by suppressing the cell cycle inhibitor p21^WAF1 ^that is induced by the sustained activation of the ERK-MAP kinase pathway [3,25]. In our study, we saw increased RhoA activity and a reduced level of constitutive p21^WAF1 ^expression in the Dlc1^gt/gt ^SFME cells. The reduced expression of Dlc1 and p21^WAF1 ^in the Dlc1 deficient cell lines was positively correlated. It is possible that activation of the Rho pathway through mutation of the Dlc RhoGAPs will complement the Ras oncogene in cell transformation *in vivo*.

## Conclusions

We have found that the Dlc1 gene has four transcriptional isoforms expressed under the influence of three alternative promoters. The alternative promoters are differentially methylated in mouse tissues and this relates to the differential expression of the proteins in the tissues examined. We also identified a 127 KDa Dlc1 protein that may originate from the 6.2 Kb alternative transcript. We generated a Dlc1 gt mouse that resulted in hympomorphic expression of the 6.1 Kb transcript and the 123 KDa Dlc1 protein. The homozygous gt Dlc1^gt/gt ^mouse embryos showed significant abnormalities in yolk sac and placental labyrinth vasculature suggesting an important developmental role for the 6.1 Kb isoform of Dlc1 during early embryonic development. A deficiency of this isoform resulted in increased GTP-Rho levels resulting in more stress fibre and vinculin associated focal adhesion formation. This in turn resulted in heightened cellular mobility.

## Methods

### Generation of Dlc1 *mutant *mice

An ES cell line (XE082) containing a gene trap insertion in the 1st intron of the Dlc1 locus (Figure 6A) was obtained from Bay Genomics (CA, USA). The gene trap plasmid ***pGT1Lxf ***contains the Engrailed-2 (En2) splice acceptor sequence upstream of the β-geo reporter gene, followed by SV40 polyA sequence. XE082 ES cells were injected into C57BL/6 blastocysts and the resulting chimeric mice were backcrossed to C57BL/6 to test for germ line transmission. One chimeric male mouse transmitted the Dlc1 gene trap allele through his germ line and was used in the subsequent studies.

We utilized an inverse PCR technique to localize the insertion site of gene trap vector (GenBank accession No: GS598775 (Figure 6A) [56]. Genomic DNA was digested with MseI, circularized with T4 DNA ligase and PCR amplified with a pair of diverging primers (Figure 6A/i-ii). This amplicon was then sequenced and indicated that the plasmid had integrated into the intron 1 of Dlc1 gene (Figure 6B). Based on the integration information, we designed: (1) a primer in the neomycin-resistance cassette of ***pGT1Lxf ***(5'-GATTCCACCGCCGCCTTCTATG); and (2) two intronic primers encompassing the integration point (5'-GTGTAAGTTTGCCTCCTCCTTTGG and 5' TCCTGCTGGTGTGTGTAAACAGAC). These three primers, when used in multiplex PCR, amplify a 570 bp product specific for the targeted allele and a 388 bp band for the wild type gene (Figure 

### Histological analysis and X-gal staining of the mutant embryos

Embryos from heterozygous crosses were dissected at 10.5 and 11.5 dpc and yolk sacs were used for DNA extraction and subsequent genotyping. Placentae were fixed in 10% neutral buffered formalin and sections were stained with H&E for microscopic examination. We isolated 10.5-11.5 dpc embryos from heterozygous crossings and they were fixed and stained as a whole mount in X gal as described [57].

### SFME cell cultures

Embryos from heterozygous intercross matings were harvested on 9.5 dpc, cells were dissociated by trypsinization and cultured in serum-free conditions which consists of epidermal growth factor, transferrin, high density lipoprotein, insulin and fibronectin coated plates [58]. Cells grow under these conditions do not senesce and have normal karyotypes. For MEF cultures, 9.5 dpc embryos were cultured in Alpha MEM (Gibco, Invitrogen, ON, Canada) supplemented with 10% fetal bovine serum and antibiotics. Cells were genotyped by PCR and studies were performed on wild type (Dlc^wt/wt^), heterozygous (Dlc1^wt/gt^) and homozygous (Dlc1^gt/gt^) cells prior to passage 10. All cell line specific experiments were repeated with multiple cell lines of each type obtained independently from different mouse litters.

### Western blotting and Immunohistochemistry

SFME as well as MEFs cells were grown on 150 mm plates until confluent, washed with ice-cold phosphate-buffered saline (PBS) and then lysed with radioimmunopreciptation assay buffer (150 mM sodium chloride, 1% NP-40, 0.5% sodium deoxycholate, 0.1% sodium dodecyl sulphate (SDS), 50 mM Tris, pH 8.0) supplemented with 1× protease inhibitor cocktail (S8820, Sigma, MO, USA). After sonication for 3× 5 s on ice, the cell lysates were clarified by centrifugation at 12,000 rpm in a Hettich-Mikro 20 centrifuge at 4°C. The protein concentration was estimated by Bicinchoninic acid protein assay kit (BCA1 and B9643, Sigma) according to the manufacturer's instructions. Twenty micrograms of total protein was separated on 8% SDS-polyacrylamide gel and transferred to a polyvinylidene fluoride microporous (PVDF) membrane (IPVHO9120, Millipore Corp, MA, USA). The membrane was then blocked for 1 h in 5% skimmed milk powder in TBST buffer (20 mM tris, 137 mM NaCl, 0.1% tween) and hybridized with Dlc-1 antibody (Sc32931, Santa Cruz Biotechnology, CA, USA; 1:500 dilution) over night. After washing in TBST buffer, the membrane was incubated with secondary horseradish peroxidase-conjugated anti-Rabbit antibody (1:2000 dilution; #7074, Cell Signaling Technology, MA, USA) for 1 h at room temperature. Immunoreactivity was detected using enhanced chemiluminescence (RPN2132, GE Healthcare, NJ, USA). The membrane was then stripped with 0.1 M NaOH and blotted again with anti-Actin or GAPDH antibody (A2006, Sigma, 1:1000 dilution). The western blots were scanned using a Storm 840 PhosphorImager scanner and quantified by densitometry using ImageQuant software (version 1.2; both from Molecular Dynamics, Inc, CA, USA).

Subconfluent cultures of different cell types were plated on fibronectin coated glass coverslips and were incubated for 12 h in serum-free medium prior to fixation with 2% neutral buffered formalin. Staining of actin fibres with TRITC labelled phalloidin (P1951, Sigma) was performed according the manufacturers' protocol. Focal adhesions were visualized by direct immunofluorescence using monoclonal anti-Vinculin-FITC antibody (F7053, Sigma). The preparations were examined on Axioplan 2 microscope (Carl Zeiss, Inc, ON, Canada) and an AxioCam HR charge-coupled device (Carl Zeiss). A 63×/1.4 oil objective lens (Carl Zeiss) was used at acquisition times of 200-300 ms for FITC (focal adhesions) 100-150 ms for TRITC (actin fibres), and 20 - 50 ms for DAPI (nuclei).

For stress fibre and focal adhesion quantification, six high resolution Z stacks images of phalloidin and antivinculin stained cells were taken at 1 μm intervals and projected as a single image after deconvolution using Axiovision 3.1 software (Zeiss). The deconvoluted flattened image was then analysed using ImageJ 1.38× public domain software. The stress fibre and focal adhesion frequency was calculated as described by Stachan *et al*. [59].

### Rho activation and *in situ *Rho GTP affinity assay

Rho activity in wild type, heterozygous and homozygous cell lines were analysed using the active Rho-GTP pull down assay. A plasmid expressing GST-Rhotekin Rho-binding domain peptide (kindly provided by Dr Alan Hall, Sloan-Kettering Institute, New York, USA) was transformed into BL21 cells and expression was induced with 0.5 mM isopropyl β-D-1 thiogalactopyranoside for 2 h at 37°C. GST fusion proteins were purified on glutathione-Sepharose 4B beads (GE Healthcare Bio-Sciences Inc, Quebec, Canada).

SFME cells were grown in 150 mm dishes and were then deprived of growth factors for 12 h followed by addition of lysophosphatidic acid (LPA, 20 ng/mL) for 30 min. At the end of the treatment, cells were rinsed with ice-cold PBS and 500 μL of lysate buffer was added to each dish. Cells were scraped, and cell lysates were clarified at 10,000 × g for 10 min. Pull-down assays were performed according to Ren and Schwartz [60] with 30-40 μg of GST-fusion protein and 800-1500 μg of protein lysate per sample.

*In situ *detection of active Rho was performed as described by Berdeaux *et al*. [54,61]. Cells growing on glass coverslips were briefly washed in PBS and immediately fixed with freshly prepared 4% paraformaldehyde (PFA) in PBS for 10 min at 4°C. After 30-min of incubation in PBS containing 3% BSA, 0.1 M glycine, and 0.05% Triton X-100 followed by incubation with the soluble GST-RBD (30 μg/ml) or GST (reduced) for 2 h at 4°C, the samples were washed three times and incubated with an anti-GST monoclonal antibody (1:1000 dilution) in wash buffer for 1 h at room temperature. The signal was detected by incubation with FITC conjugated anti-rabbit IgG (1:1000 dilution) for 45 min at room temperature. Samples were mounted in antifade reagent containing DAPI.

### RNA isolation and cDNA preparation

RNA was isolated from different tissues of C57BL/6J mouse, as well as SFME cell lines using TRIzol Reagent (Invitrogen Inc, ON, Canada) according to the manufacturer's instructions. Total RNA (5 μg) was reversed transcribed using SuperScript II Reverse transcriptase (Invitrogen) and random hexamers (Invitrogen).

### 3'rapid amplification of cDNA ends (RACE) analysis

The 3'RACE were carried out using DNase treated total RNA extracted from the cerebellum of 12.5 dpc embryo, placenta, adult brain and heart of C57BL/6J mice. First strand cDNA synthesis for 3' end was performed using 50 ng of the oligonucleotide dT Primer (Table 1), 5 μg of total RNA and SuperScript II reverse transcriptase (Invitrogen) following the manufacturer's protocol [62]. The 3'end was then amplified using a primer that contains part of the oligonucleotide dT primer, which binds to these cDNAs at their 3' ends, and exon specific primers from the Dlc1 gene (Table 1). A second amplification series was then performed using internal nested primers (Table 1) to suppress the amplification of non-specific products.

**Table 1:** List of primers used for 3'RACE, real time polymerase chain reaction (PCR), multiplex PCR, genotyping, and northern probe preparation. All primer sequences are in 5'-3' direction.

Primers for 3'RACE			
1st strand CDNA synthesis	CCAGTGAGCAGAGTGACGAGGACTCGAGCTCAAGCTTTTTTTTTTTTTTTTT		

	**Forward primers**	**Reverse primers**	**Sequencing primers**

1st round PCR	CTATCAGAAAGAGGAGCTGGGAAG AGACCGAACAGCCTTTTATTTCTG ACGTCTTAGCAGCTCCTTATGTAG ACAAAAGCACTCTCGGGGTCAG AAACTGGACCAACTAGACCAAGAC ATCTGAATTGCACAGGCAAAACAG GCCCTAAGCCTTTCCAGATG CTGCGCCGACCTTAATGTGTAG	CCAGTGAGCAGAGTGACG	

2nd round PCR	CTATCAGAAAGAGGAGCTGGGAAG AGACCGAACAGCCTTTTATTTCTG ACGTCTTAGCAGCTCCTTATGTAG ACAAAAGCACTCTCGGGGTCAG AAACTGGACCAACTAGACCAAGAC ATCTGAATTGCACAGGCAAAACAG TCCTTCAGCGACCACATCAGAG CGCCGACCTTAATGTGTAGAGAC	GAGGACTCGAGCTCAAGC	ATTTTTTTTTTTTTTTTTTTTT GTTTTTTTTTTTTTTTTTTTTT CTTTTTTTTTTTTTTTTTTTTT & different forward primers

**Primers for Northern probe**			

1st round PCR	CTGCGCCGACCTTAATGTGTAG	GTTGCAGTCACGGGTGCTTC	

2nd round PCR	CTCGAGCTGCGCCGACCTTAATGTGTAG	GTCGACGTTGCAGTCACGGGTGCTTC	

**Primers for real time quantification of splice variants**			

7.6 Kb transcript	TCCACCGATCCATCATCCACTC	CGGTGAGGACTGATCTCCAGCTTCATG	

6.2 Kb transcript	TCCTTCAGCGACCACATCAGAG	GACTGGTTTCCCCCAGTATGCA	
		
6.1 Kb transcript	CGCCGACCTTAATGTGTAGAGAC		

2.1 Kb transcript	TCCACCGATCCATCATCCACTC	GCCCACTGAGTGCTGAGACTG AAGTAC	

GAPDH	TTC CGT GTT CCT ACC CCC AAT GTGT	GGAGTTGCTGTTGAAGTCGCAGGAG	

**Primers for methylation study**			

6.1 Kb Transcript	Biotin-GTAAGAAAAGGTAGAGGGGAAATTGAGTA	CTCCTTCCCCCTTTCCTAAAATAT	TTTTCCCAAAATCTACACT

6.2 Kb transcript	TAGGAGGTTAGTATGGGTTGTA	Biotin-AAACCTAATACTAACATAAAACAATATC	AAATTGTTTTTTTGATTTTAA

**Multiplex PCR primers for genotyping Dlc1 gene trap embryos**			

	TCCTGCTGGTGTGTGTAAACAGAC	GTGTAAGTTTGCCTCCTCCTTTGG	

	GATTCCACCGCCGCCTTCTATG	TCAAGCAAAAAGGTCTCGTATGTTGTG	

**Multiplex PCR primers for fusion transcripts**			

(7.6+2.1)Kb transcript	TCCACCGATCCATCATCCACTC	ACGACGACAGTATCGGCCTCAG	
		
6.2 Kb transcript	TCCTTCAGCGACCACATCAGAG		
		
6.1 Kb transcript	CGCCGACCTTAATGTGTAGAGAC		

All 3'RACE reactions were electrophoresed through 1.5% agarose gels and visualized. 3'RACE products were excised from the agarose gel, purified using Qiagen gel extraction kit (Qiagen Inc, ON, Canada) and cloned into the pGEM-T Easy Vector Systems (Promega Fisher Scientific, ON, Canada) using the manufacturer's instructions.

After cloning 3'RACE products, both strands of DNA were sequenced for each construct. Sequencing was performed by the DNA Sequencing facility at Manitoba Institute of Cell Biology using BIG DYE terminators from Applied Biosystems, Inc (PE Applied Biosystems, ON, Canada) and sequencing reactions were run on ABI 310 DNA Sequencer.

### Northern blot analysis of DLC-1 mRNA

Poly(A)^+ ^RNA was prepared from different tissues of C57BL/6J mice and SFME cell lines using mRNA isolation kit (Roche Diagnostics, Quebec, Canada) and analysed by Northern blot hybridization using 2.2% (w/v) acrylamide-0.5% (w/v) agarose gel [63]. The blot was probed with a ^32^P-labelled 3.3 Kb RNA probe generated from Dlc1 6.1 Kb isoform. The 3.3 Kb probe was generated by nested PCR primers (Table 1) and cloned into the Xho1 restriction site of Litmas28i vector (New England BioLabs, MA, USA) containing T7 promoter on both sides. The vector was linearized and the radiolabelled antisense RNA probe was generated using T7 polymerase.

### Quantification of Dlc1 isoforms by real time and multiplex PCR

The relative expression of the different transcriptional variants was determined by quantitative real time and multiplex RT-PCR. Three forward primers were designed, one primer each from the exon 1 of 6.1 and 6.2 Kb transcripts and the third primer designed from the exon 4 of 7.6 Kb transcript, which is also common to 2.1 Kb transcript. The 6.1 and 6.2 Kb transcript specific forward primers were combined with a reverse primer designed from exon 2 and the 7.6 Kb transcript specific forward primer was combined with a reverse primer specific to exon 4 of 6.1 Kb transcript, respectively. PCR primers were designed using the Gene Fisher-version 2 software (Bielefeld University Bioinformatics Service, Bielefeld, Germany) for quantification of the various isoform variants (listed in Table 1). Absolute quantification of the transcripts was performed in an iCyclerIQ (Biorad, ON, Canada). Each reaction mix contained SYBR Green (Sigma S9430), Fluorescein calibration dye (Biorad, 170-8780) and 200 nM of each primer, 200 nM dNTP solutions, 1 × Reaction buffer, 1.5 mM MgCl_2 _and 0.5 U Taq polymerase. The thermal cycling conditions were: 95°C for 1 min, 40 cycles of 95°C for 15 s, 62°-65°C for 30 s and 72°C for 30 s. In order to determine the absolute copy number of target transcripts, Dlc1 isoforms (6.1, 6.2 and 7.6 Kb) and GAPDH cDNAs were amplified using specific primer sets as described in Table 1. The purified PCR products were cloned in pGEM-T easy vector system and the plasmid DNA was used to generate calibration curves, which also permitted evaluation of PCR efficiencies using the formula *E *= 10^[-1/slope]^. The efficiency of amplification was checked for all targets by performing a series of serial dilutions of template for each primer pair in triplicate. Real time PCR data quality control calculations were run on the SAS program as described [64,65]. The E value varied from -2.05 to -3.4 which represents an acceptable PCR efficiency between 90%-95% [66]. Quantitative RT- PCRs were then conducted for different mouse tissue samples, and the specificity of each amplified product was confirmed by the melting curve analysis (Figure 3A). Results were expressed as copy number of specific isoforms per 1000 copies of GAPDH cDNA (Figure 3B).

For multiplex PCR, we used two different strategies. First, the different isoforms were quantified relative to GAPDH internal standard and second, all the splice variants were quantified to the corresponding fusion transcript that was generated by fusion to the βgeo gene, which is part of the gene trap insertion. Relative expression of cDNA equivalent of 400 ng total RNA was quantified using two sets of primers in the same PCR reaction; one set specific to the Dlc1 isoform and the other set specific to either the GAPDH gene or to the βgeo fusion cDNA. The PCR products were run in a 1.5% agarose gel and stained with ethidium bromide for visualization.

### *In vitro *scratch assay

Cell migration rates of wild type, heterozygous and homozygous cell lines were compared by an *in vitro *scratch assay [67]. Prior to the migration experiments, 60 mm cell culture plates were incubated for 4 h with 10 mg/mL fibronectin in Alpha-MEM. Coated dishes were blocked with 3 mL of 2 mg/mL bovine serum albumin (BSA) for 1 h at 37°C. Then, excess fibronectin was removed by washing with PBS and dishes were refilled with 3-5 mL of media before plating the cells. The wild type, heterozygous and homozygous fibroblast batches were seeded onto the fibronectin coated plates and cultured until confluent. After reaching confluency, the cell monolayer was scratched with a p200 pipette tip in two diagonal lines. The cell debris was removed and the edge of the scratch was smoothened by washing the cells once with 1 mL of the growth medium and then replaced with 5 mL of medium and incubated at 37°C for 18 h. In order to obtain the same field during the image acquisition, reference points were created by marking the underneath of the dish with a fine pointed marker using a stereo-microscope. Subsequent photographs of the scratches were taken at 0, 6 and 18 h time intervals using a phase contrast microscope. We quantified the cell migration rate by determining the total area that the MEF cells moved from the edge of the scratch toward the center of the scratch during 0-6 h and 6-18 h of incubation following the creation of the wound in terms of pixels calculated manually using the Image J 1.38× public domain software (available at http://rsb.info.nih.gov/ij/).

### Methylation study of the Dlc1 promoter region

Chromosomal DNA was isolated from different mouse tissues by standard phenol chloroform extraction and ethanol precipitation [68]. Five micrograms of genomic DNA was then treated with bisulphite reagent for conversion of unmethylated cytosines of genomic DNA to uracils as previously described [68]. An aliquot of converted DNA (~100-300 ng) was used for amplification of the promoters of the Dlc1 6.1 and 6.2 Kb transcripts. The PCR primers were designed from the regions of the promoter that do not harbour any CpG dinucleotides (Table 1, Figure 5A). Primers were designed using PSQ Assay design software version 1.0.6 (Biotage) and one of the primer was biotinylated to generate single stranded DNA which was then sequenced using specific sequencing primers and the Pyromark gold reagent (5× 96 PSQ 96 MA) in Biotage Pyromark Q96 MD machine. The methylation pattern was analysed using PyroQ-CpG software version 1.0.0.

### Statistical analysis

The one-way analysis of variance (ANOVA) test was used to compare three different cell lines with respect to the mean of Dlc1 protein expression, P21^WAF1 ^protein expression, focal adhesion frequency, stress fibre frequency and the cell migration rate. Two-way ANOVA was used to assess the effect of LPA on active Rho induction in three different cell lines compared to controls. Correlation between Dlc1 and P21^WAF1 ^protein expression was analysed using Pearson correlation test. All the statistical analysis was performed using Sigma Stat 7 for Windows software, version 2.03, 1997, SPSS Inc, IL, USA. Statistical significance was defined as *P *< 0.05.

## Abbreviations

cDNA: complimentary DNA; Dlc: deleted in liver cancer; dpc: days post coitum; ES: embryonic stem; H&E: haematoxylin and eosin; gt: gene trapped; MEF: mouse embryonic fibroblast; PBS: phosphate-buffered saline; PCR: polymerase chain reaction; RBC: red blood cells; RNA: ribonucleic acid; ROCK: rho-associated coiled coil forming kinase; RT-PCR: reverse transcription PCR; SAM: sterile alpha motif; SDS: sodium dodecyl sulphate; SFME: serum-free mouse embryo; START: StAR-related liquid transfer.

## Authors' contributions

MGS carried out inverse PCR, genotyping of the transgenic mice, analysis of embryos, molecular analysis of RNA transcripts, protein expression studies, fluorescence IHC, promoter methylation analysis, *in vitro *scratch assay, designing of the study, analysis and the drafting of the manuscript. SL was participated in analysis of the transgenic mouse embryos. YG participated in genotyping of the transgenic mice. NW was involved in generation of the transgenic mice. CB participated in protein expression studies. GGH conceived the study and supervised generation of the transgenic mice. MM conceived and supervised the study, analysed the data, drafted the manuscript.
